# Supplementary material for: Coordinated Transcriptional Waves Define the Inflammatory Response of Primary Microglial Culture
Source: Int J Mol Sci. 2023 Jun 30;24(13):10928. doi: 10.3390/ijms241310928 (PMC10341870; doi:10.3390/ijms241310928)
Supplement: Supplementary file 1 [file ijms-24-10928-s001.zip › Figures S1-S7.pdf]

Supplemental Materials

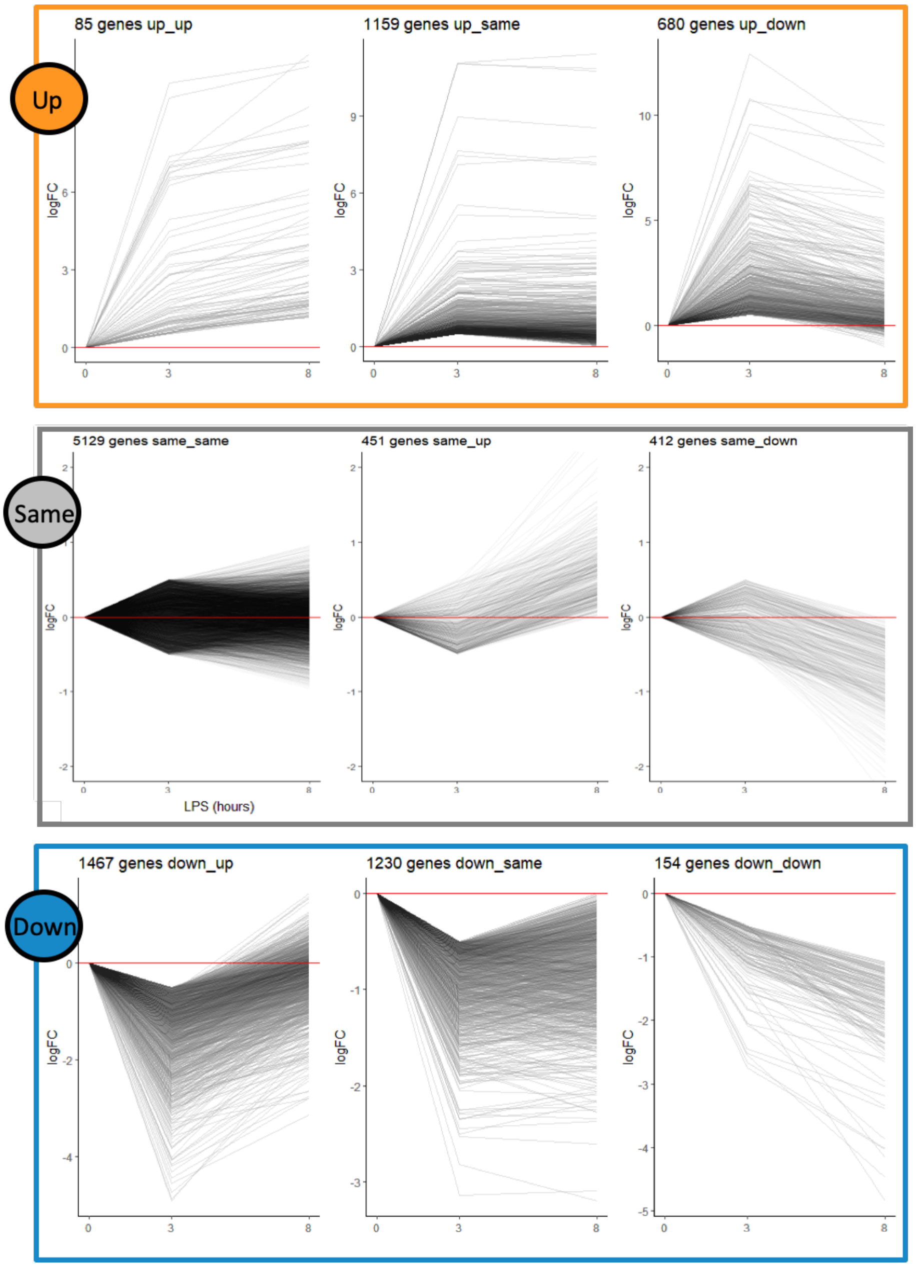

Figure S1. Gene expression trends on naïve, not treated microglial cells and following activation with bzATP/LPS for 3 hrs and 8 hrs (x-axis). Each differentially expressed gene is shown as a gray line connecting the hours from the exposure. The y-axis shows the log(base 2) fold change (log FC). Note that the scale of the y-axis changes according to the range of the genes in the clustered group. The number of genes associated with each cluster group (Marked as Up, Same and Down) are indicated. The horizontal red line indicates a baseline with no change in expression.

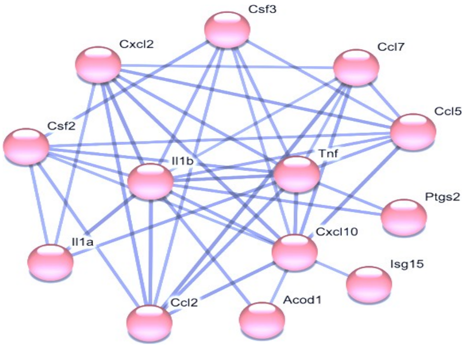

Figure S2. A connectivity map according to STRING protein interaction network. Only the genes (13/25) with a high confidence connection score (>0.9) are shown. The entire list of 25 top DE genes are presented in Figure 5.

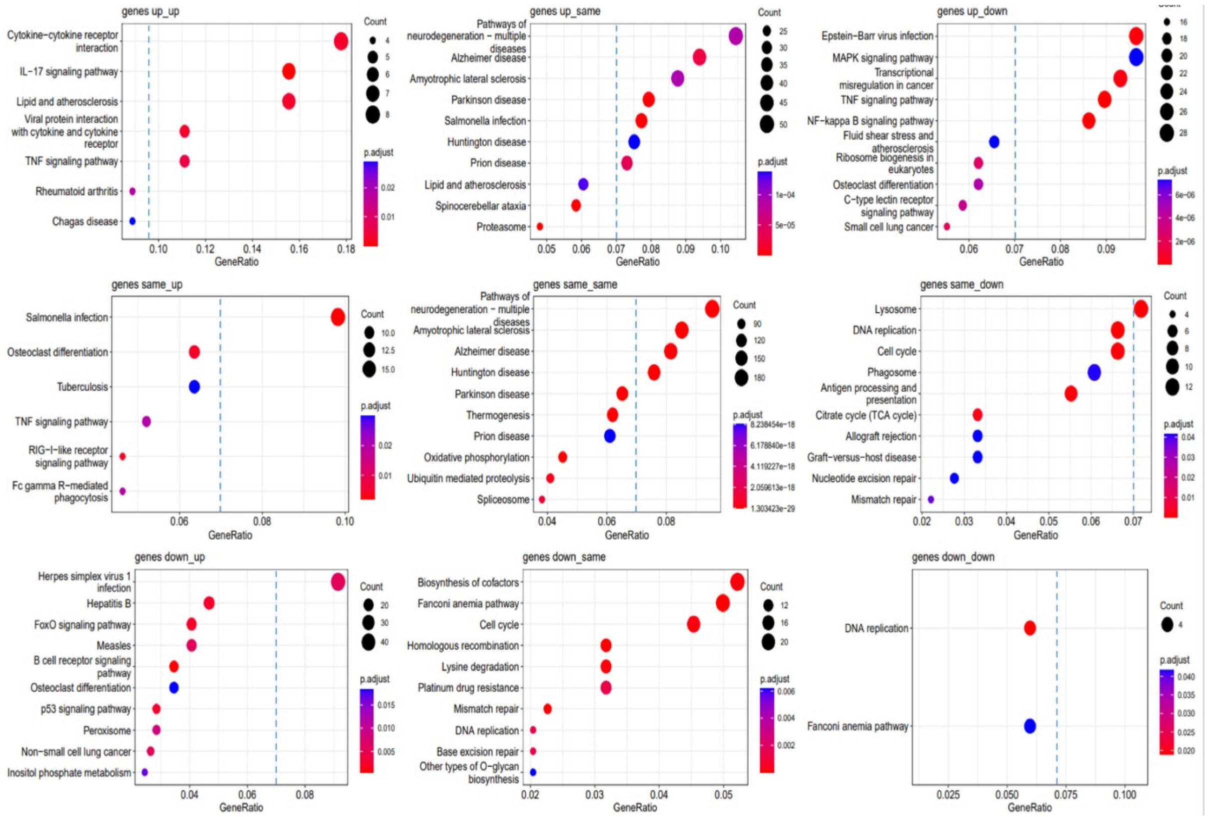

Figure S3. Enrichment analysis for all 9 differentially expressed (DE) gene clusters followed bzATP/LTP induction. Enrichment analysis for all DE genes clusters. Top, middle and bottom panels are associated with Up, Same and Down set (3 hrs). Annotations are according to KEGG pathway resource. Statisticalenrichment is depicted by p-adjust and significance is bounded to FDR <0.05. The size of the dots captures the number of proteins. Note that the enrichment significance scale, the number of genes associated with each enriched pathway, and the gene ratio (i.e., fraction of genes in the cluster that are included in the specific pathway) differ among gene cluster. A vertical dashed line marks Gene ratio = 0.7 for better visual alignment.

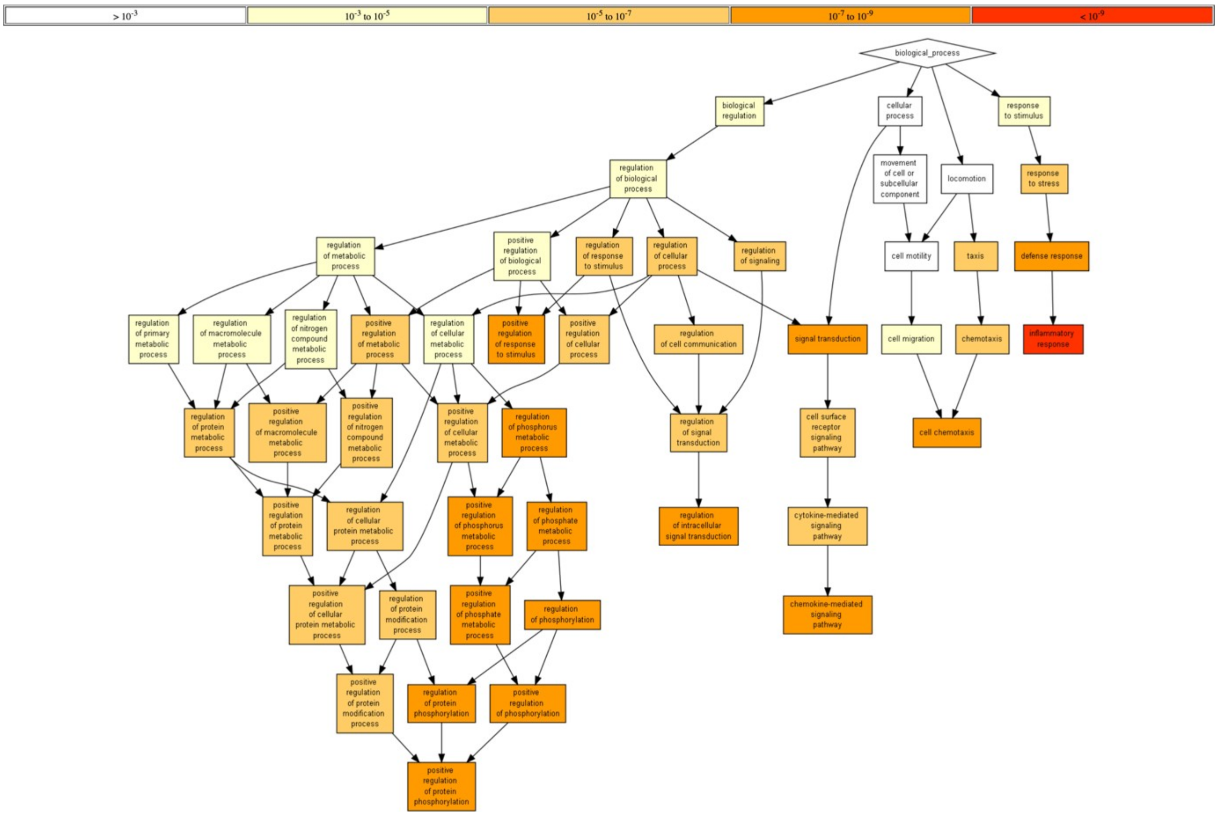

Figure S4. Enrichment analysis for gene ontology (GO) of upregulated DE genes. An enrichment test was applied on 585 statistically significant DE genes that were strongly induced (>1.5-fold increase in expression relative to that of non-treated cells, and average expression level >10 TMM). The test was based on hypergeometric analysis, followed by multiple testing corrections, (GOrilla statistical tool). Results shown are for biological process. Only GO terms with p-value <1.0e-07 were included in the analysis. The color scale (white to red) with the associated range of p-values is shown (Top).

A

pathway	description	false discovery rate
WP3632	Lung fibrosis	0.00096
WP2573	Primary focal segmental glomer	0.0108
WP2432	Spinal cord injury	0.0067
WP493	Mapk signaling pathway	0.0067

B

pathway	description	false discovery rate
WP246	TNF-alpha NF-kB signaling pathw	5.48e-09
WP3632	Lung fibrosis	2.03e-08
WP222	Cytokines and inflammatory resp	3.77e-06
WP37	IL-1 signaling pathway	1.01e-05
WP2432	Spinal cord injury	1.01e-05
WP373	IL-3 signaling pathway	1.01e-05
WP1496	Oxidative damage response	1.49e-05
WP1254	Apoptosis	2.56e-05
WP493	Mapk signaling pathway	0.00015
WP447	Adipogenesis genes	0.00050
WP1243	EBV LMP1 signaling	0.00067
WP572	EGFR1 signaling pathway	0.0023
WP2292	Chemokine signaling pathway	0.0025
WP1560	MicroRNAs in cardiomyocyte hyp	0.0053
WP1763	PluriNetWork: mechanisms assoc	0.0059
WP113	TGF-beta signaling pathway	0.0105

Figure S5. Results show the significant enriched pathways from WikiPathways at a false discovery rate (FDR) <1.0e-2. **(A)** bzATP (8 hrs) and **(B)** bzATP/LPS (8 hrs). The analysis is based on STRING network for the 100 most significant genes (sorted by DE analysis).

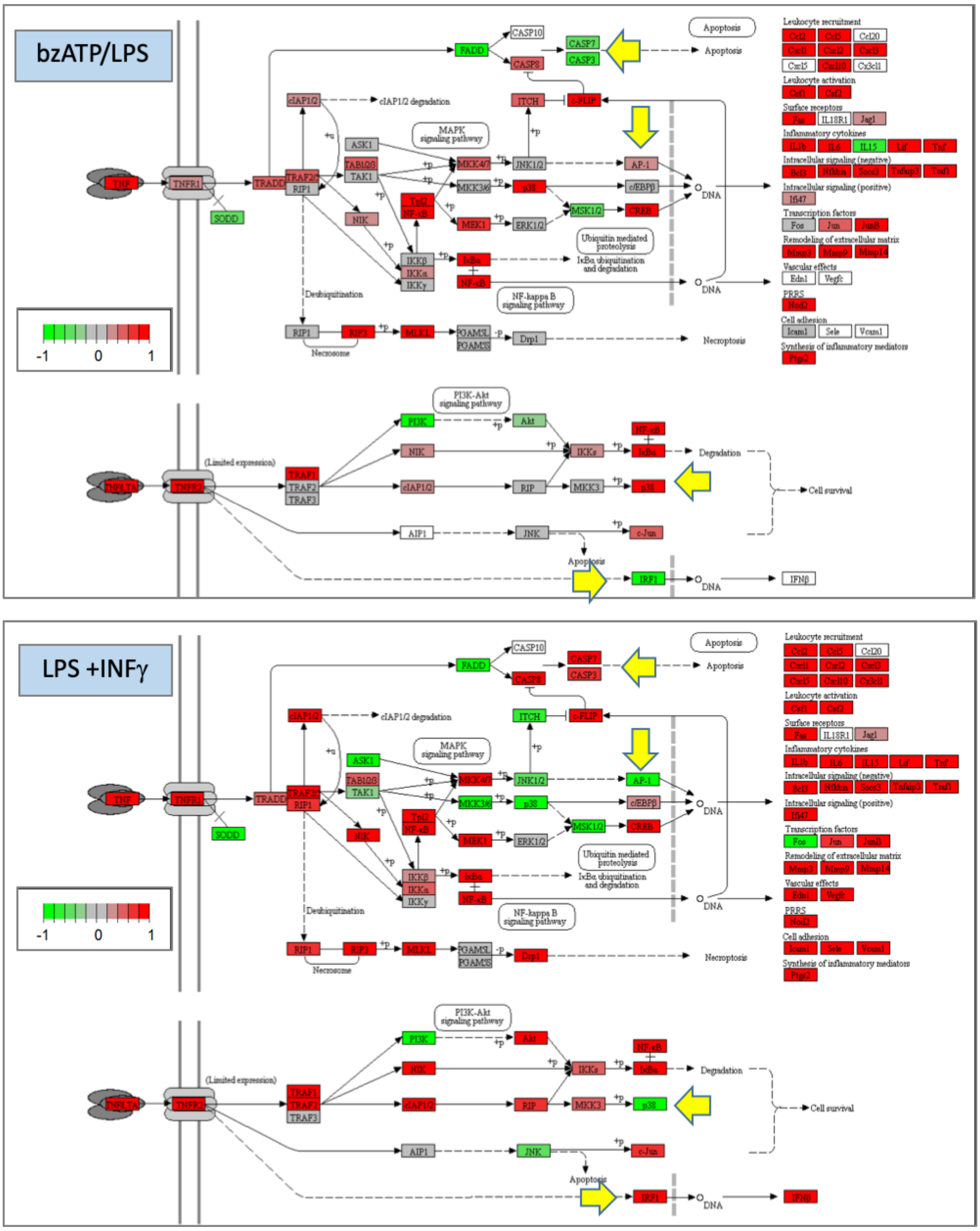

Figure S6. DE gene pattern on the KEGG pathway map of TNF signaling. The top panel indicates the genes participating in the TNF signaling. Microglia culture exposed to bzATP/LPS (8 hrs, Top), and LPS+IFNγ for 6 hrs (Bottom) are shown. The genes are colored according to by their expression trends. The colors indicate genes that are downregulated (green), upregulated (red), unchanged (gray), and undetected (white) with respect to the expression in untreated culture. Selected genes for which expression trends between the two settings of microglia activation substantially differ are marked with yellow arrows.

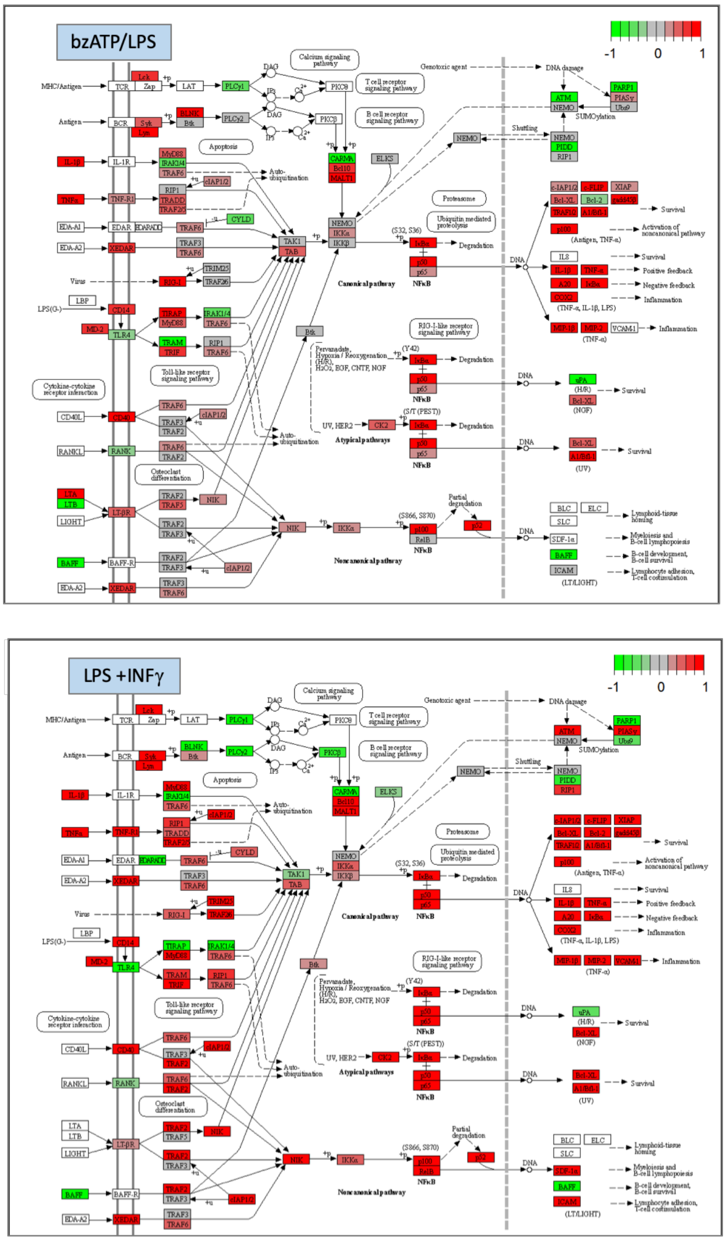

Figure S7. DE gene pattern on the KEGG pathway map of NF-kB signaling. The top panel indicates the genes participating in the NF-B signaling. Microglia culture exposed to bzATP/LPS (8 hrs, Top), and LPS+IFNγ for 6 hrs (Bottom) are shown. The genes are colored according to by their expression trends. The colors indicate genes that are downregulated (green), upregulated (red), unchanged (gray), and undetected (white) with respect to the expression in untreated culture.
